# Can the Natural Diversity of Quorum-Sensing Advance Synthetic Biology?

**DOI:** 10.3389/fbioe.2015.00030

**Published:** 2015-03-10

**Authors:** René Michele Davis, Ryan Yue Muller, Karmella Ann Haynes

**Affiliations:** ^1^Ira A. Fulton School of Biological and Health Systems Engineering, Arizona State University, Tempe, AZ, USA; ^2^Biological Design Graduate Program, Arizona State University, Tempe, AZ, USA; ^3^Department of Chemistry and Biochemistry, Arizona State University, Tempe, AZ, USA; ^4^School of Life Sciences, Arizona State University, Tempe, AZ, USA

**Keywords:** quorum sensing, homoserine lactone, crosstalk, orthogonal, genetic wire, synthetic gene circuit

## Abstract

Quorum-sensing networks enable bacteria to sense and respond to chemical signals produced by neighboring bacteria. They are widespread: over 100 morphologically and genetically distinct species of eubacteria are known to use quorum sensing to control gene expression. This diversity suggests the potential to use natural protein variants to engineer parallel, input-specific, cell–cell communication pathways. However, only three distinct signaling pathways, Lux, Las, and Rhl, have been adapted for and broadly used in engineered systems. The paucity of unique quorum-sensing systems and their propensity for crosstalk limits the usefulness of our current quorum-sensing toolkit. This review discusses the need for more signaling pathways, roadblocks to using multiple pathways in parallel, and strategies for expanding the quorum-sensing toolbox for synthetic biology.

## Modules from Natural Quorum-Sensing Networks Can be Decoupled and Integrated into Synthetic Systems

Scientists first explored the genetic circuitry of a quorum-sensing system through basic research of *Vibrio fischeri*, a symbiotic microbe that populates the light organ of the bobtail squid, *Euprymna scolopes*. Researchers identified an operon called “Lux” that allowed individual *V. fischeri* cells to produce a glowing phenotype by expressing Luciferase specifically in dense bacterial populations (Ruby and Nealson, 1976). Explorations of other microbial genomes revealed dozens of Lux homologs that are collectively known as homoserine lactone (HSL) quorum-sensing networks (Fuqua et al., 1996; Williams et al., 2007; Dickschat, 2010). In addition to bioluminescence, they found that these bacteria use quorum sensing to couple population density with the onset of group behaviors such as virulence, biofilm formation, sporulation, competence, and disruption of neighboring bacterial biofilms (Eberl, 1999).

Homoserine lactones networks are more commonly known as *N*-acyl homoserine lactone (AHL) quorum-sensing networks. However, our discussion includes LuxI-like synthases that produce compounds with a homoserine lactone ring but groups other than the acyl tail. In this review, we will consider homoserine lactone, HSL, to include AHLs as well as non-acyl tail compounds. We will refer to HSL with an acyl tail as acyl-HSL.

**Figure 1:**
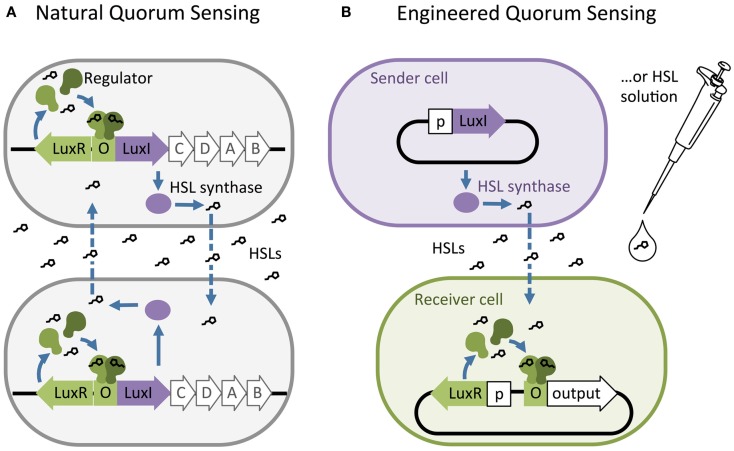
**The structure of natural and artificial homoserine lactone (HSL) networks**. **(A)** Natural HSL quorum-sensing networks such as the luciferase expression system in *Vibrio fischeri* regulate the expression of gene clusters (e.g., LuxR, I, C, D, A, and B). **(B)** Quorum-sensing networks have been decoupled and used to build engineered, synthetic systems to control the expression of any gene of interest (output). O = “Operator” binding site for the regulator protein, p = constitutive promoter.

Homoserine lactones quorum-sensing networks generally consist of an HSL synthase LuxI-like protein, an HSL-binding LuxR-like regulator, and promoters that are regulated by LuxR-like/HSL complexes. The LuxI-like HSL synthase enzyme produces chemical signals called HSLs (Engebrecht and Silverman, 1984; Kaplan and Greenberg, 1987). Most HSLs diffuse passively across the cell membrane, while some require active transport. Quorum sensing is triggered when high external HSL concentrations drive net influx, allowing HSLs to bind and activate a LuxR-like regulator. The activated LuxR-like/HSL complex binds to a 20 base pair inverted repeat known as a Lux-like-box and regulates expression of downstream genes (Figure 1A). Synthases from various species of bacteria produce different HSL signals, and their corresponding regulators generally bind their cognate HSL with varying levels of specificity.

Researchers have also identified two other families of cell–cell communication networks: autoinducer-2 (AI-2) networks (Vendeville et al., 2005; Reading and Sperandio, 2006) and autoinducing peptides (AIPs), also called peptide pheromone networks (Kleerebezem et al., 1997; Reading and Sperandio, 2006). While AI-2 and AIP networks may be used in engineered systems, the molecular components of HSL networks are simpler, more diverse, and require little modification to function as expected when they are transferred into new host cells. These characteristics of the HSL family of quorum-sensing networks are well suited for building sophisticated, multi-component, synthetic systems. Therefore, we focus primarily on the HSL networks in this review.

**Figure 2:**
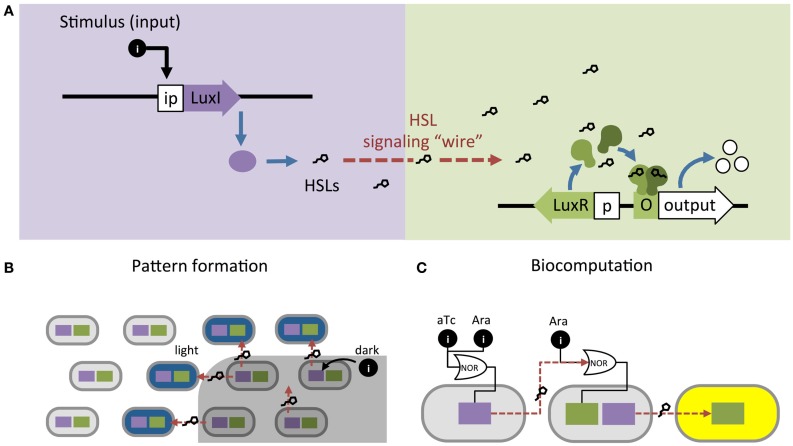
**Homoserine lactone (HSL)-based genetic wiring supports the function of sophisticated synthetic systems**. **(A)** Quorum-sensing pathways used as wires to link an input stimulus with expression of an output gene. **(B)** An edge detector expressed as Light Λ Neighboring Dark. The absence of light (dark) allows HSL production to activate LacZ (blue) in neighboring light-exposed cells (Tabor et al., 2009). **(C)** Biocomputational systems implement complex Boolean expressions by linking combinations of small molecule inputs (e.g., tetracycline aTc and arabinose Ara) to outputs (e.g., yellow fluorescence) using HSL signaling (Tamsir et al., 2011).

Synthetic biologists and genetic engineers often use HSL quorum-sensing pathways to engineer novel behaviors in prokaryotic microorganisms. In these engineered systems, quorum-sensing pathways are used as a set of decoupled components where the HSL synthase is the “Sender” component and the regulator and promoter are collectively the “Receiver” component (Figure 1B) (Miller and Bassler, 2001). They can be employed as “genetic wires” linking the functional elements of multi-component biological systems (Tamsir et al., 2011; Goñi-Moreno et al., 2013). The wires connect circuit components within a cell or across a population of single or multiple strains. The Sender converts an input stimulus into a transmittable signal, the HSL, which activates the Receiver. The Receiver modulates expression of an output as designated by the designer (Figure 2A). This input stimulus may be anything that activates a promoter, including heavy metals (Prindle et al., 2012a; Wang et al., 2013), specific wavelengths of light (Tabor et al., 2009), biochemical signals secreted by pathogens (Saeidi et al., 2011; Gupta et al., 2013), the hypoxic microenvironment surrounding a tumor (Anderson et al., 2006), and HSLs from tandem quorum-sensing networks (Tamsir et al., 2011). The output may be any gene controlled by a Lux-like promoter, such as a visible reporter (Canton et al., 2008; Tabor et al., 2009; Tamsir et al., 2011), cell motility (Liu et al., 2011), antimicrobial proteins (Saeidi et al., 2011; Gupta et al., 2013), and anti-cancer drugs (Anderson et al., 2006).

The simplicity of these networks allows researchers to model how quorum-sensing-controlled gene expression is regulated in response to HSL signal concentration (McMillen et al., 2002; Pai and You, 2009; Pai et al., 2014). These models can inform how a quorum-sensing network should be implemented in a synthetic circuit to achieve the desired behavior (McMillen et al., 2002; Pai and You, 2009). Furthermore, dry lab researchers have used modeling to demonstrate how quorum-sensing systems control group response in the presence of noisy signal concentrations, supporting their use in synthetic biology as robust circuit components (Koseska et al., 2009; Weber and Buceta, 2013).

Incorporating quorum-sensing networks into production strains has advanced the field of metabolic engineering. Quorum sensing has been used to synchronize gene expression across a population to reduce cell-to-cell variability and to increase yields in engineered strains (Danino et al., 2010; Prindle et al., 2012a,b; Anesiadis et al., 2013). For example, by linking a Lux-based genetic oscillator with a gas phase signal oscillator, researchers coordinated gene expression among 2.5 million cells across 5 mm of space with minimal noise (Danino et al., 2010; Prindle et al., 2012a,b). Anesiadis et al. (2013) employed this type of circuit in a production strain, where they engineered a cell-density-dependent switch using the Lux system to control production of serine in an *Escherichia coli* knockout strain. Group-controlled gene expression implemented by an HSL quorum-sensing network leads to overall higher serine production.

Quorum-sensing networks are also used in genetic circuits to perform computation. Tabor et al. (2009) took advantage of the diffusibility of HSL through agar to build a bacterial edge detector using the Lux network. They demonstrated that stationary physical spacing of bacteria relative to different inputs drives controlled expression of an output. The circuit was designed such that bacteria exposed to darkness expressed HSLs but no output (LacZ). The circuit allowed only bacteria that were both adjacent to HSL-producers and exposed to light to express LacZ, which resulted in a pigmented outline at the edges of a light-masked region (Figure 2B). While most biocomputation is digital, Daniel et al. (2013) showed the versatility of quorum-sensing networks by demonstrating analog computation using the Lux network; their circuit converts logarithmic HSL input into linear fluorescent output over a large range of HSL concentrations. Thus far, engineered biocomputation has used monolayers of cells. Three-dimensional (3D) colony-printing techniques will increase the sophistication of these systems (Connell et al., 2013). Controlled spacing of colonies based on HSL-diffusion rates could allow engineering a temporal element into a split circuit.

In the preceding examples, the cells in each system are expressing the same circuit. However, engineers may also coordinate gene circuits distributed among multiple populations. Brenner et al. (2007) used the Rhl and Las networks from *Pseudomonas aeruginosa* to build two strains of *E. coli* that form a biofilm together once both populations reach a threshold density. Balagaddé et al. (2008) used components from the Lux and Las networks to engineer a predator–prey relationship between *E. coli* strains. High predator population density induces cell death in the prey strain, while high prey population density supports survival of the predator strain. You et al. (2004) placed a cell death gene under the control of the Lux promoter and built a bistable system that maintains a population density of a defined range. At high cell density, the Lux network activates a cell death. At decreased cell density, the cell death gene is inactive and the population begins to grow again. Computation may be split across multiple strains, distributing the energy demands of a complex computation that are too great for a single cell (Ji et al., 2013; Payne and You, 2014) (Figure 2A). Wang et al. (2013) built a two-strain, three-input biosensor in *E. coli* that produces RFP only in the presence of three heavy metal contaminants: arsenic, mercury, and copper. Cell 1 produces LuxI after exposure to arsenic and mercury; Cell 2 expresses RFP in response to 3O-C6-HSL produced by Cell 1 and copper. Tamsir et al. (2011) linked circuits expressed in multiple cell populations using two quorum-sensing networks derived from *P. aeruginosa*, Rhl and Las (Figure 2C). They implemented complex Boolean expressions using different spatial arrangements on agar plates. Their system is built with the functionally completed NOR operator and can implement any Boolean expression.

## Crosstalk between Quorum-Sensing Pathways Challenges the Development of Synthetic Genetic Circuits

Attempts to isolate, study, and apply quorum-sensing pathways for bioengineering is often thwarted by unexpected crosstalk. Quorum sensing is a popular tool among synthetic biologists for designing multicellular systems, but widely utilized HSL quorum-sensing networks are currently limited to three pathways: Lux, Las, and Rhl. These networks all exhibit crosstalk with each other, complicating the design of complex genetic systems implemented with quorum-sensing networks.

For instance, a single regulator can be activated by multiple acyl-HSL-class molecules, resulting in cross-activation of regulators from different species of bacteria. This phenomenon was observed in a proof-of-concept system designed by Canton et al. (2008) wherein an output gene for green fluorescent protein (GFP) was placed under the control of a LuxR receiver module (Wu et al., 2014). Four chemically distinct acyl-HSLs, C6-HSL, C7-HSL, 3O-C8-HSL and, at higher concentrations, C8-HSL, all activated expression of GFP at levels comparable to or even greater than the cognate LuxI acyl-HSL, 3O-C6-HSL (Canton et al., 2008). Many different HSL synthases, including EsaI, ExpI, and AhlI, produce the same major cognate acyl-HSL as LuxI, suggesting that these pathways would have high levels of crosstalk if built into the same network (Miller and Bassler, 2001; Põllumaa et al., 2012). In the report of their predator–prey, two-strain system, Balagaddé et al. (2008) discussed low-level crosstalk between LuxI and LasR, which was recently confirmed by observing LuxI and LasR interactions in a single-strain system (Wu et al., 2014). However, Balagadde’s system functioned such that crosstalk was apparently below the threshold for altering intended behavior. Interestingly, this type of crosstalk is also observed in nature (Fuqua et al., 1996). For example, two opportunistic pathogens, *Burkholderia cepacia* and *P. aeruginosa*, are known to co-infect patients with cystic fibrosis (Lewenza et al., 2002). Each pathogen’s quorum-sensing regulators respond to the other’s HSLs, resulting in coordination of virulence-gene expression.

Crosstalk can also occur at the level of the target, or “output,” gene; similarities between promoter sequences and the DNA-binding domains within the regulator proteins contribute to crosstalk between quorum-sensing pathways. The acyl-HSL-activated LuxR regulator stimulates transcription at its cognate promoter as well as the Esa promoter, while acyl-HSL-activated LasR, EsaR, and ExpR regulators are also capable of initiating transcription at the Lux promoter (von Bodman et al., 2003; Saeidi et al., 2011; Shong et al., 2013). While this type of crosstalk can be avoided by using only one regulator per strain, they will not behave as two orthogonal wires within a single cell.

## Expanding the Set of Orthogonal Quorum-Sensing Pathways Enables Design of Complex Genetic Circuits

Synthetic circuits may be engineered to detect specific combinations of input signals so long as each sensing pathway functions independently (orthogonally) without undesired intercommunication (crosstalk). Genetic circuits designed to respond to complex combinations of environmental conditions must distinguish and integrate multiple distinct input signals. Orthogonal quorum-sensing pathways are necessary to implement complex circuits that respond to signals produced by living cells, rather than requiring synthetic, exogenous inputs. Engineered division of labor is a major research area in metabolic engineering (Bernstein et al., 2012; Vinuselvi and Lee, 2012); orthogonal quorum-sensing modules will enable further development of cell-autonomous metabolic regulation in multi-strain bioreactor systems. Quorum-sensing circuits could be used to engineer multi-strain, self-monitoring microbial populations that perform energetically expensive metabolic processes in a single culture. Multiple co-cultured strains could be designed to monitor and maintain a target population ratio, or steps in a metabolic process could be timed for accumulation of precursors (Tamsir et al., 2011).

Circuit sophistication is limited by metabolic capacity, transcription and translation resources, and crosstalk within the cell. Moon et al. (2012) pushed the bounds of single-cell computational capability by building a four input AND gate in *E. coli*. Their complex logic gate allows living bacterial cells to express GFP in the presence of four exogenous compounds and no fewer. Transcription activator complexes were decoupled and placed under the control of distinct inducible promoters that respond to the presence of soluble compounds (arabinose, IPTG, tetracycline, and the acyl-HSL 3O-C6-homoserine lactone) in the cell culture medium. More complex circuits could be implemented by replacing the exogenous inputs in the Moon et al. system with quorum-sensing wires linking cells performing independent computation. Scaling can be achieved through modularity by building complex computational systems with simple independent components. By designing the components in separate strains and connecting them with orthogonal quorum-sensing wires, computational steps can be performed independently without exhausting cellular resources.

Connecting complex circuits requires orthogonal HSL networks to independently signal each strain’s computation. However, using even two quorum-sensing networks in parallel is constrained by crosstalk. To our knowledge, there is no published demonstration of three or more orthogonal quorum-sensing networks in a single system. The complexity of multi-input integration circuits remains constrained by reliance on exogenous signals and by the limited number of orthogonal input–output pathways.

## Strategies for Minimizing Crosstalk

Promiscuous interactions between HSLs and regulators, as well as between regulators and promoters, prevent many quorum-sensing systems from operating independently and in parallel. Some have used gene-network engineering approaches to mitigate crosstalk (Brenner et al., 2007; Balagaddé et al., 2008; Tamsir et al., 2011; Wu et al., 2014). For instance, Brenner et al. (2007) engineered their system to avoid crosstalk between the Las and Rhl networks. They split the networks between two strains to eliminate promoter–regulator crosstalk and controlled HSL synthase production via a positive feedback loop to achieve a two-strain, biofilm-forming consortium.

Another approach to eliminate crosstalk between signaling pathways is using quorum-sensing pathways from distinct families (the aforementioned HSL, AI-2, and AIP pathways). Significant variance in the chemistry of the signaling molecules suggests that cross-reactivity is unlikely: HSLs contain a lactone ring with a hydrocarbon acyl or aryl tail, AI-2 is a furanosyl borate diester composed of two five-membered rings stabilized by a boron atom, and AIPs are relatively large circular peptides composed of amino acids (Chen et al., 2002; Marchand and Collins, 2013). However, this approach may be limited in its flexibility since both AI-2 and AIP require active transport and multiple proteins to generate and detect the signals. With a few exceptions, HSL networks require only two proteins and one promoter. While AI-2 quorum sensing is limited to only one signaling molecule, multiple AIP pathways may exist that do not have cross-reactivity. Marchand and Collins (2013) recently demonstrated modularity and orthogonality of two AIP signals from *Staphylococcus aureus*. In their system, *E. coli* was the AIP sender, producing and secreting two AIPs, and two engineered strains of *Bacillus megaterium* each received one of the signals but not the other. While the ability to use two AIPs in a single cell was not explored, this is a promising result and further research could demonstrate orthogonality between AIPs and HSL quorum sensing.

Directed evolution could also be used to generate regulator proteins that specifically respond to any desired HSL. Mutational analyses and 3D protein structure data have helped to identify key amino acid residues that govern the interaction between regulators and acyl-HSL ligands. Using positive and negative selection, Collins et al. (2006) generated a LuxR mutant that no longer responds to the cognate 3O-C6-HSL but gained responsiveness to C10-HSL, to which wild-type LuxR does not respond. They then demonstrated the orthogonality of LuxR wild type versus the mutant. However, directed evolution of regulator proteins to generate novel orthogonality is technically daunting and only generates mutants with minor changes to the wild-type binding pocket, limiting the range of possible novel behaviors. Furthermore, they bind to and activate the same promoter and, while this feature could be leveraged to build OR gates, they cannot be used as orthogonal networks in the same cell without further mutagenesis to alter promoter-binding specificity.

Finally, scientists could explore other microbial genomes for quorum-sensing homologs that have not yet been exploited for synthetic biology. Comparative genomics has identified dozens of HSL family (Lux-like) homologs in divergent species (Case et al., 2008). A major advantage of exploring wild-type homologs over directed evolution is that natural evolution has already “discovered” functional regulators in a very broad exploration space of amino acid sequences. Evolution has selected for regulator proteins of significantly different sizes, as opposed to artificial selection techniques that, due to practical constraints, do not deviate significantly from pre-existing primary structures.

## The Basis of Specificity in the HSL Signaling Family

Investigations of microbial quorum-sensing pathways have revealed molecular characteristics that underlie the diversity of HSL signaling pathways in different species. These signaling pathways have been distinguished on the basis of the operator binding sites at promoters elsewhere (Vannini et al., 2002). In this review, we focus on diversity in the geometries of HSL signaling molecules and the HSL-binding pockets within the regulator proteins.

**Figure 3:**
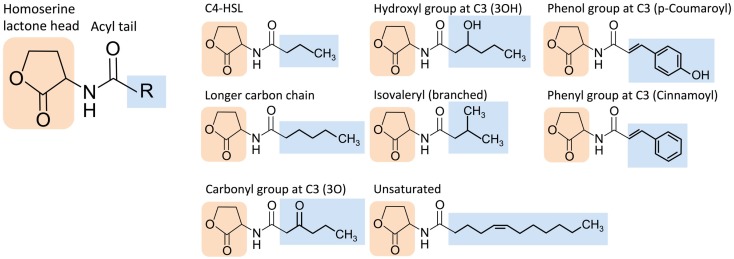
**Structural diversity of homoserine lactone (HSL) signaling molecules that are produced by bacteria**. Representative variants are shown.

**Figure 4:**
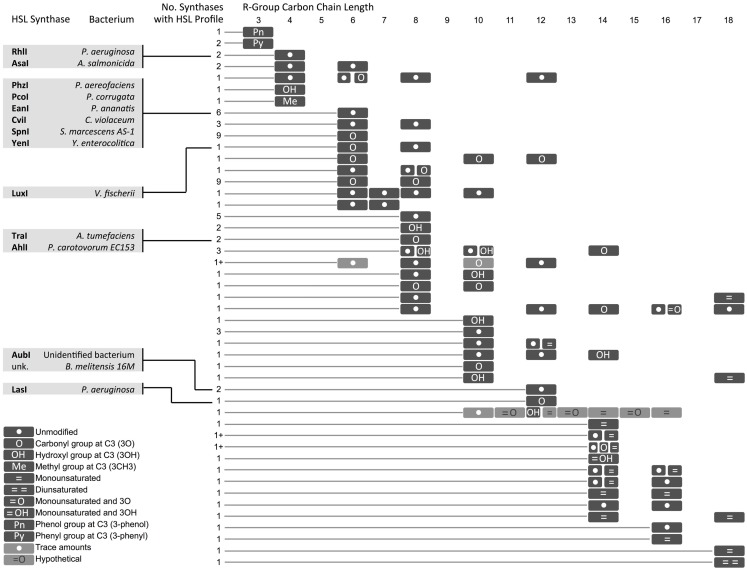
**Diversity in homoserine lactones (HSLs) produced by synthases across bacterial species**. Select synthases and representative species are shown in gray boxes. Each row represents one or several HSLs produced by one or more synthase. The number of synthases with each HSL profile is indicated. A (1+) indicates that the exact number of synthases is not known. A comprehensive chart with referenced literature is provided as Supplemental Material. Hypothetical = cases where one of two molecules with identical mass [i.e., 3O-C(*n*)-homoserine lactone (HSL) and C(*n* + 1)-HSL] might be produced by the bacterium, but the molecules could not be resolved with mass spectrometry. *The hypothetical monounsaturated C12-HSL produced by AbaI is not shown here.

The extensive molecular diversity of naturally occurring HSL signaling molecules suggests that many functionally distinct HSLs, and thus orthogonal pathways, may exist. HSLs vary in the R-group, an acyl or aryl tail that extends from a homoserine lactone head (Dickschat, 2010) (Figure 3). HSL synthases have been reported to generate HSLs of varying carbon chain lengths, branching functional groups, and hydrocarbon saturation (Figure 4). Straight-chain acyl R-groups vary by chain lengths (e.g., C4-HSL versus C6-HSL in Figure 3) from 4 to 18 carbon atoms. Some acyl R-groups carry side-group replacements at the third or fourth carbon in the chain: a carbonyl group at C3 (e.g., 3O-C6-HSL), a hydroxyl group at C4 (e.g., 3OH-C6-HSL), or a methyl group at C3 (e.g., branched-chain isovaleryl-HSL). Aryl R-groups have a phenol group at C4 (p-Coumaroyl-HSL), or a phenyl group at C4 (Cinnamoyl-HSL). R-groups also differ by the degree of saturation in the carbon chain (e.g., monounsaturated 3OH-C14:1); unsaturation results in a carbon–carbon double bond, which changes the shape of the acyl tail, compared to the saturated form.

In some instances, a single synthase can produce two or more HSL variants. This variety arises from the species-specific combination of acyl tails that are carried into the HSL synthesis pathway by acyl-carrier proteins (ACPs) or aryl-CoA (Lindemann et al., 2011). Some HSL synthases display promiscuity in ACP or CoA-binding affinity and can catalyze formation of several different HSL molecules. Other HSL molecules show no overlap across species, suggesting that the cognate regulators may have evolved to respond specifically to certain HSLs, and orthogonal quorum-sensing systems may exist in nature.

**Figure 5:**
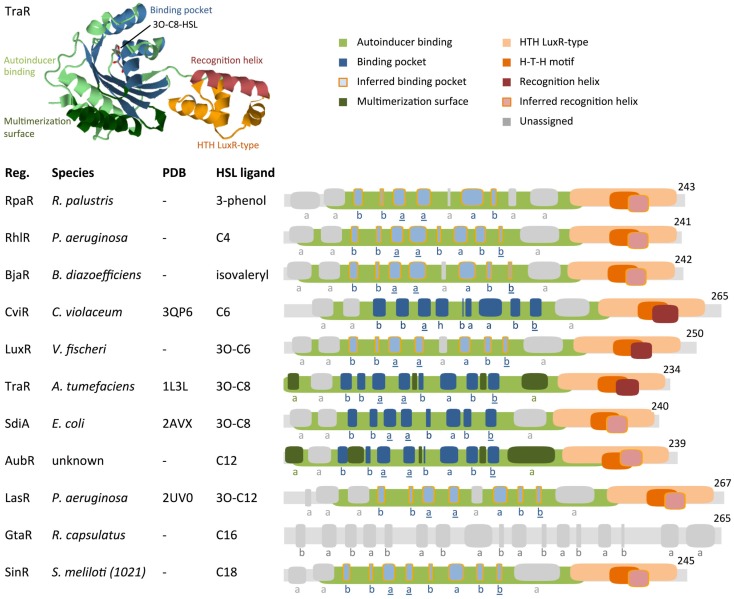
**Comparison of protein motifs in select regulators**. The three-dimensional (3D) structure of TraR is shown as an example of how domains and the homoserine lactone (HSL) ligand are typically positioned in space. The underlined letters in the b–b–a–a–b–a–b–b secondary structure motif indicate the location of highly conserved amino acids that form hydrogen bonds with the homoserine lactone head of HSLs. Published 3D structure data (Protein Data Bank) are listed where available (“–” = not available). Abbreviations used are: Reg. = regulator protein, H–T–H = helix–turn–helix, a = alpha helix, b = beta strand, h = 3/10 helix. Database entries for conserved motifs are: autoinducer binding = IPR005143, HTH LuxR-type = PS50043. Inferred binding pockets are patterns of secondary structures that are similar to the TraR binding pocket. Inferred recognition helices are the second alpha helix from the C-terminus. Secondary structures for proteins with no available 3D structure data were mapped using the Jpred prediction tool (Cole et al., 2008). Maps were generated using DomainDraw (Fink and Hamilton, 2007).

Regulator proteins from the HSL quorum-sensing family (LuxR homologs) consist of two major domains: an N-terminal autoinducer (HSL) binding region and a C-terminal region that binds DNA (Figure 5). To visually compare the topologies of functional regions in different regulators, we have generated scaled protein domain maps using descriptions from the literature (Egland and Greenberg, 2001; Zhang et al., 2002; Bottomley et al., 2007; Chen et al., 2011) and annotations from protein domain-scanning databases Uniprot (UniProt Consortium, 2014), Prosite (Sigrist et al., 2013), InterPro (Mitchell et al., 2015), and the Protein Data Bank (Berman et al., 2000) (Figure 5). Autoinducer-binding regions (InterPro IPR005143) contain roughly six alpha helices and six beta strands. Published 3D structures for TraR (PDB 1L3L) (Zhang et al., 2002), LasR (PDB et al., 2UV0) (Bottomley et al., 2007), CviR (PDB 3QP6) (Chen et al., 2011), and SdiA (Yao et al., 2006) reveal that a five-strand beta sheet is sandwiched between two three-helix bundles. The C-terminal DNA-binding domains are characterized as “helix–turn–helix” (HTH) regions (Prosite PS50043) that consist of four alpha helices. The second and third helices within the HTH region are often identified as a conserved H–T–H motif (Prosite PRU00411); the third helix has been characterized as the DNA recognition helix in TraR (Zhang et al., 2002). When HSL molecules bind to their corresponding quorum-sensing regulator, they often induce multimerization of regulator proteins. This multimeric state is the active form, capable of binding an inverted DNA sequence repeat at the target promoter and inducing transcription of downstream genes.

Analysis of the HSL-regulator binding pockets suggests that the shape, size, hydrophobicity, and functionalization determine the binding affinity of a regulator for a specific HSL. This implies that comparison of known HSL–regulator interactions may identify likely candidates for orthogonal quorum-sensing networks. For example, it has been hypothesized that quorum-sensing systems that produce long, straight-chain acyl-HSLs have regulators with longer binding pockets; likewise, a system that uses acyl-HSL molecules with branching functional groups will have regulators with binding pockets that accommodate the branches (Bottomley et al., 2007). Thus, taking sterics into account, a quorum-sensing system that uses HSL molecules with a relatively short hydrocarbon tail and bulky functional groups may be orthogonal to a system that uses long-chain, non-branched HSL molecules.

Hydrophobic interactions between the HSL tail and amino acid residues within the binding pocket suggest that these binding interactions are dominated by van der Waals forces (Bottomley et al., 2007) (Figure 3). Because the HSL tail is buried within the binding pocket, the hydrophobicity of each component also determines the entropic stability, with a predominantly hydrophobic tail pairing stably with a predominantly hydrophobic binding pocket and less hydrophobic tail pairing stably with a less hydrophobic binding pocket. Therefore, HSL tail and binding pocket hydrophobicity may be a predictor of orthogonality between quorum-sensing pathways.

Pharmacophore models for HSL-regulator binding developed by Geske et al. (2008) support the idea that functionalization of the HSL molecule underlies binding pocket selectivity. Their models are based on the response of Tra, Las, and Lux regulators to libraries of HSLs and synthetic analogs in a system that used beta-galactosidase as the output gene. Comparison of the atomic geometries of ligands reveals three general properties linked with HSL efficacy: spacing of hydrophobic regions, hydrogen bond donor regions, and hydrogen bond acceptor regions within the R-group attached to the lactone ring. For instance, TraR shows the greatest response to a group of ligands in which the acyl tail contains one hydrogen bond donor region followed by two hydrogen bond acceptor regions arranged in *trans* and ended in a hydrophobic region (Geske et al., 2008).

Conservation and divergence in the conformation of regulator N-terminal HSL-binding regions support the idea that variation in HSL R-groups coordinates selective regulator–ligand interactions. Here, we explore whether motifs in the protein structures of regulators provide insight into the underlying mechanism of HSL-binding selectivity. Primary sequence alignments show 10–25% identity in regulator homologs (Bottomley et al., 2007) and therefore provide very limited information. We have attempted a more coarse-grained approach on a select set of well-characterized regulators by annotating secondary structures that correspond to the TraR binding pocket. For regulators that lack published 3D structure data, we have annotated secondary structures as hypothetical HSL-binding pockets (Figure 5).

The autoinducer-binding region contains two functional domains in its tertiary structure: the multimerization surface and the HSL-binding pocket. The multimerization surface of the TraR homodimer consists primarily of alpha helices a1 and a6 (Bottomley et al., 2007), plus other residues within loops that link helices and beta strands (Figure 5). The HSL-binding pocket binds a single HSL molecule in the space between a five-strand antiparallel beta sheet and a three-helix bundle (Bottomley et al., 2007). In the primary structure of TraR, these secondary structures are arranged in the order of b–b–a–a–b–a–b–b. The first and second alpha helix and the last beta strand of this motif (underlined in Figure 5) contain the amino acids that form hydrogen bonds with the homoserine lactone head of the HSL ligand. These residues are highly conserved in LuxR protein homologs, reflecting a common binding mechanism at the non-variable head regions of HSL molecules. In contrast, the variable acyl tail extends into the region of the binding pocket that is formed by residues that show less conservation in LuxR homologs, suggesting a mechanism for HSL selectivity (Bottomley et al., 2007).

TraR and SdiA are most responsive to the ligand 3O-C8-HSL (Michael et al., 2001; Geske et al., 2008). These regulators contain the same b–b–a–a–b–a–b–b pattern of secondary structures in their HSL-binding pocket domains (Figure 5). This pair of regulators fits the attractive idea that binding pockets with similar secondary structures may prefer the same HSL ligands. However, comparisons of other regulators challenge this idea. While some regulators that respond to HSLs with smaller or larger R-groups deviate from the b–b–a–a–b–a–b–b motif, there are others, i.e., RhlR, LasR, and SinR, which contain the same motif yet respond to different ligands: C4-HSL, 3O-C12-HSL, and C18-HSL, respectively (Llamas et al., 2004; Kumari et al., 2006; Geske et al., 2008). It is possible that variations in specific residues in RhlR, LasR, and SinR account for their preferences for different ligands. AubR contains a substitution of the third beta strand with an alpha helix in the b–b–a–a–b–a–b–b motif, similar to LuxR and BjaR. LuxR and BjaR respond to 3O-C6-HSL (Canton et al., 2008) and isovaleryl-HSL (Lindemann et al., 2011), respectively. Assuming that the ligand for AubR is C12-HSL [produced by AubI (Nasuno et al., 2012)], AubR, LuxR, and BjaR represent another set of regulators where similarities in secondary structures do not appear to correspond to similar ligands. Exploration of the range of HSL-responsiveness of these regulators may provide more insight into their structure–function relationships.

Interestingly, no HSL-regulator protein-related motifs appear in GtaR. Leung et al. (2012) reported that GtaR regulates its target promoter (a Lux promoter homolog) in response to C16-HSL and cell-free growth medium collected from HSL-producing strains. GatR shows sequence conservation with the TatD family of deoxyribonuclease proteins. Like the LuxR homologs, TatD proteins contain interspersed beta strands and alpha helices. Here, we have annotated predicted secondary structures within GtaR; these domains are inferred from comparisons of GatR with closely related TatD proteins that have published 3D structures. Given its distinct arrangement of secondary structures, GtaR might represent a unique class of HSL-responsive regulator proteins.

## Conclusion and Discussion

The information we present here on the diversity of HSL molecules and regulator proteins is insufficient to conclude that the structures of regulator binding pockets and the atomic geometries of the HSL ligands imply orthogonality. Regulators that respond to distinct HSL ligands show different protein folding patterns in some cases but similar structures in others. With limited data on regulator promiscuity, secondary structure alone cannot predict HSL ligand preference; thus, interaction between specific amino acid residues and atoms within the HSL molecule may need to be considered. This investigation is limited by the lack of 3D structure data for LuxR homologs.

Many gaps in knowledge remain in understanding the extent of orthogonality or interaction between the homologous pathways in living cells. To date, the published functional studies of the quorum-sensing homologs in synthetic circuits (HSL synthases, regulators, and promoters) include just three homologous quorum-sensing pathways, or they use purified compounds to stimulate one or a few regulators. Furthermore, the available 3D structure data for regulator proteins is sparse compared to the total number of putative regulator homologs that have been identified via metagenomic analysis (Nasuno et al., 2012). More comprehensive analyses to study the responses of regulator proteins to different HSLs, such as that of Geske et al. (2008), may enable us to predict and select orthogonal pathways for use in complex synthetic systems. For instance, *E. coli* could be used as a universal host to carry dozens of decoupled sender and receiver components (Figure 1), derived from the genomes of various bacterial species. Culture media from sender strains could be used to stimulate receiver strains carrying a reporter driven by a receiver system (regulator protein and its corresponding promoter).

The discovery of novel orthogonal quorum-sensing pathways will provide metabolic engineers and synthetic biologists with HSL signaling wires that do not cross-react. Using these insulated, independently functioning pathways, synthetic circuits could be designed to detect distinct combinations of multiple input signals and scale simple single-cell components to sophisticated multi-strain circuits. It is imperative to continue research on quorum-sensing pathway behavior across multiple disciplines, including crystallography, molecular biology, microbiology, metabolic engineering, and synthetic biology, to fill critical gaps in knowledge that have prevented us from engineering highly sophisticated biological systems.

## Conflict of Interest Statement

The authors declare that the research was conducted in the absence of any commercial or financial relationships that could be construed as a potential conflict of interest.

## Supplementary Material

The Supplementary Material for this article can be found online at http://www.frontiersin.org/Journal/10.3389/fbioe.2015.00030/abstract
