# Corrigendum: Can the Natural Diversity of Quorum-Sensing Advance Synthetic Biology?

**DOI:** 10.3389/fbioe.2015.00099

**Published:** 2015-07-07

**Authors:** René Michele Davis, Ryan Yue Muller, Karmella Ann Haynes

**Affiliations:** ^1^Ira A. Fulton School of Biological and Health Systems Engineering, Arizona State University, Tempe, AZ, USA; ^2^Biological Design Graduate Program, Arizona State University, Tempe, AZ, USA; ^3^Department of Chemistry and Biochemistry, Arizona State University, Tempe, AZ, USA; ^4^School of Life Sciences, Arizona State University, Tempe, AZ, USA

**Keywords:** quorum sensing, homoserine lactone, crosstalk, orthogonal, genetic wire, synthetic gene circuit

The gene WP_023917333 was incorrectly used to generate the GtaR protein motif map in Figure 5 of the original publication, which led us to publish erroneous conclusions about GtaR structure and function (Davis et al., 2015). At the time this manuscript was published, the gene WP_023917333 was incorrectly titled “LuxR family transcriptional regulator *Rhodobacter capsulatus*” in the NCBI database. Analysis of the correct GtaR protein sequence (WP_013066073) does not show “sequence conservation with TatD family of deoxyribonuclease proteins” nor does it lead us to conclude that GtaR “might represent a unique class of HSL-responsive regulator proteins” (Davis et al., 2015).

**Figure 5:**
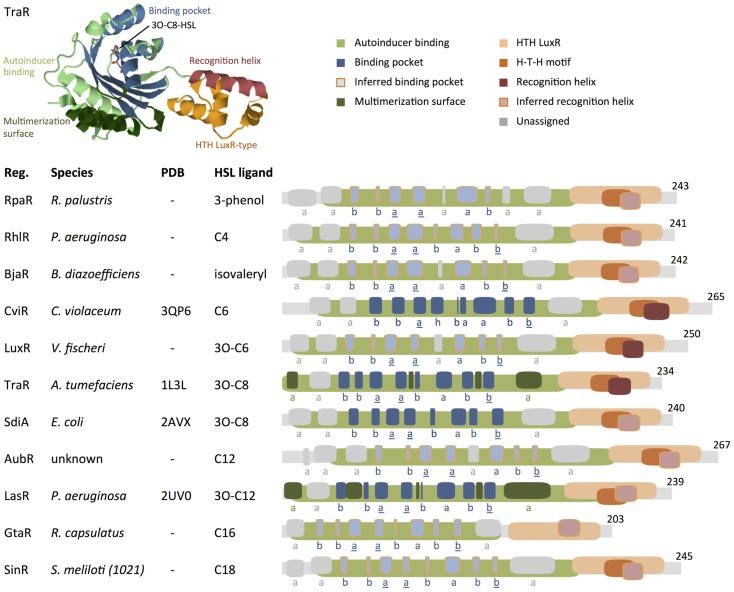
**Comparison of protein motifs in select regulators**. The three-dimensional (3D) structure of TraR is shown as an example of how domains and the homoserine lactone (HSL) ligands are typically positioned in space. The underlined letters in the b–b–a–a–b–a–b–b secondary structure motif indicate the location of highly conserved amino acids that form hydrogen bonds with the homoserine lactone head of HSLs. Published 3D structure data (Protein Data Bank) are listed where available (“–” = not available). Abbreviations used are: Reg. = regulator protein, H–T–H = helix–turn–helix, a = alpha helix, b = beta strand, h = 3/10 helix. Database entries for conserved motifs are: autoinducer binding = IPR005143 (Mitchell et al., 2015), HTH LuxR = SM00421 (Schultz et al., 1998; Letunic et al., 2015). Inferred binding pockets are patterns of secondary structures that are similar to the TraR-binding pocket. Inferred recognition helices are the second alpha helix from the C-terminus. Secondary structures for proteins with no available 3D structure data were mapped using the Jpred prediction tool (Cole et al., 2008). Maps were generated using DomainDraw (Fink and Hamilton, 2007). Figure modified from Davis et al. (2015).

Analysis of the correct protein sequence (WP_013066073) shows that GtaR contains the same b–b–a–a–b–a–b–b motif as RhlR, LasR, and SinR. All four regulator proteins, RhlR, LasR, GtaR, and SinR, respond to different ligands: C4-HSL, 3O-C12-HSL, C16-HSL, and C18-HSL, respectively (Llamas et al., 2004; Kumari et al., 2006; Geske et al., 2008; Leung et al., 2012). We surmise that variations in specific residues may account for the regulator proteins’ preferences for different ligands.

The GtaR C-terminus does not match the HTH LuxR-type motif (Prosite PS50043) originally annotated in Figure 5 but does match an “HTH_LuxR” DNA-binding motif designated as SMART motif SM00421 (Schultz et al., 1998; Letunic et al., 2015) at amino acids 140–197. This motif is present in all the regulators analyzed. Figure 5 now illustrates HTH LuxR regions (SMART SM00421) instead of PS50043. Furthermore, in the original publication, the protein motif maps were switched between LasR and AubR, and the SidA map was scaled incorrectly. We have corrected these errors in a new version of Figure 5.

## Conflict of Interest Statement

The authors declare that the research was conducted in the absence of any commercial or financial relationships that could be construed as a potential conflict of interest.
