# Supplementary material for: Can the Natural Diversity of Quorum-Sensing Advance Synthetic Biology?
Source: Front Bioeng Biotechnol. 2015 Mar 10;3:30. doi: 10.3389/fbioe.2015.00030 (PMC4354409; doi:10.3389/fbioe.2015.00030)
Supplement: Supplementary file 1 [file Table_1.PDF]

- • Unmodified
- O Carbonyl group at C3 (3O)
- OH Hydroxyl group at C3 (3OH)
- Me Methyl group at C3 (3CH3)
- = Monounsaturated
- == Diunsaturated
- =O Monounsaturated and 3O
- =OH Monounsaturated and 3OH
- Pn Phenol group at C3 (3-phenol)
- Py Phenyl group at C3 (3-phenyl)
- • Trace amounts (light grey)
- =O Hypothetical (light grey)

Carbon Chain Length

Species	Synthase(s)	Ref(s)	3	4	5	6	7	8	9	10	11	12	13	14	15	16	17	18
Rhodopseudomonas palustris	RpaI	Lindemann 2011	Pn															
Silicibacter pomeroyi	unknown	Schaefer 2008	Pn															
Bradyrhizobium strain BTAiL	BtaI, BraI	Lindemann 2011, Ahlgren 2011	Py															
Pseudomonas aeruginosa	RhlI	Fuqua 2002		•														
Aeromonas salmonicida	AsaI	Miller 2001		•														
A. hydrophila	ahyI	Khajanchi 2011		•		•												
Serratia sp. ATC 39006, marcescens MG1	SmaI/SwrI	Galloway 2011		•		•												
Halomonas anticariensis	HanI	Tahrioui 2011, Llamas 2005		•		• O		•				•						
Xenorhabdus nematophilus 19061	unknown	Dickschat 2010		OH														
B. japonicum	BjaI	Lindemann 2011		Me														
P. chlororaphis	PhzI	Miller 2001				•												
P. corrugata	PcoI	Licciardello 2007				•												
Pantoea ananatis	EanI	Tahrioui 2011, Morohoshi 2007				•												
Chromobacterium violaceum	CviI	Mcclean 1997				•												
Serratia marcescens AS-1	SpnI	Galloway 2011				•												
Yersinia enterocolitica	YenI	Miller 2001				•												
Burkholderia cenocepacia	CciI	Malott 2005				•		•										
Rhizobium leguminosarum	RhiI	Rodelas 1999, Schripsema 1996, Miller 2001				•		•										
Ralstonia solanacearum	SolI	Rodelas 1999, Schripsema 1996, Miller 2001				•		•										
Erwinia carotovora	ExpI	Galloway 2011				O												
P. syringae	AhlI	Dulla 2009				O												
Sodalis glossinidius	SogI	Pontes 2008				O												
P. agglomerans	EagI	Miller 2001				O												
E. chrysanthemi	ExpI	Miller 2001				O												
E. stewartii	EsaI	Miller 2001				O												
Y. pseudotuberculosis	YpsI	Miller 2001				O												
Pectobacterium carotovorum ATCC390048	CarI	Põllumaa 2012				O												
P. atrosepticum SCRI1043	ExpI	Põllumaa 2012				O												
Vibrio fischeri	LuxI	Eberhard 1981				O		•										
P. putida	PpuI	Galloway 2011, Wagner-Döbler 2005				O				O		O						
Azospirillum lipoferum B518	AlpI	Vial 2006				•		• O										
P. carotovorum SCC3193	ExpI	Põllumaa 2012				O		O										
P. carotovorum 71	AhlI	Põllumaa 2012				O		O										
Burkholderia sp. CBMB40	unknown	Dickschat 2010				•	•	•		•								
E. psidii	unknown	Dickschat 2010				•	•											
B. cepacia	CepI	Lewenza 1999						•										
B. thailandensis	BtaI1	Chandler 2009						•										
S. liquefaciens	SwrI	Miller 2001						•										
Y. pseudotuberculosis	YtbI	Miller 2001						•										
Roseobacter litoralis DSM 7001T	unknown	Wagner-Döbler 2005						•										
B. thailandensis	BtaI3	Chandler 2009						OH										
B. phytofirmans PsJN	unknown	Dickschat 2010						OH										
Agrobacterium tumefaciens	TraI	Moré 1996						O										
P. carotovorum EC153	AhlI	Põllumaa 2012						O										
B. pseudomallei	bpmI 1, 2, 3	Ulrich 2004						• OH		• OH				O				
B. vietnamiensis	uncharacterized	Conway 2002				• (light grey)		•		O (light grey)		•						
A. lipoferum TVV3	unknown	Vial 2006						•		OH								
A. lipoferum TVV3	AlpI	Vial 2006						O		O								
Dinoroseobacter shibae DFL 16	unknown	Wagner-Döbler 2005						•										=
Sinorhizobium meliloti Rm1021	SinI	Marketon 2002						•				•		O		• =O		•
B. thailandensis	BtaI2	Chandler 2009								OH								
B. vietnamiensis	BviI	Conway 2002								•								
A. lipoferum TVV3	unknown	Vial 2006								•								
B. pseudomallei 008	unknown	Dickschat 2010								•								
Gymnopilus intermedius NCI1051	GinI	Iida 2008								•		• =						
Rhodospirillum rubrum	unknown	Mastroleo 2013								•		•		OH				
V. anguillarum	VanI	Miller 2001								O								
R. gallaeciencis T5	unknown	Wagner-Döbler 2005								OH								=
Unidentified soil bacterium	AubI	Nasuno 2012										•						
Brucella melitensis 16M	unknown	Dickschat 2010										•						
P. aeruginosa	LasI	Fuqua 2002										O						
Acinetobacter baumannii	AbaI	Niu 2008								• (light grey)	=O (light grey)	OH, = (light grey)	=O (light grey)	= (light grey)	=O (light grey)	= (light grey)		
R. sphaeroides	CerI	Miller 2001												=				
Roseovarius tolerans EL 52, 78, 83, 90, 164, 171, 172T, 222	unknown(s)	Wagner-Döbler 2005												• =				
R. tolerans EL 78, 90	unknown(s)	Wagner-Döbler 2005												• O =				
R. leguminosarum bv. viciae 8401	CinI	Miller 2001, Dickschat 2010												=OH				
R. tolerans EL 164	unknown	Wagner-Döbler 2005												• =		• =		
R. tolerans EL 171	unknown	Wagner-Döbler 2005												• =		•		
Jannaschia helgolandensis HEL 10T, 26	unknown	Wagner-Döbler 2005												=		=		
R. capsulatus	GtaI	Schaefer 2002												•		•		
R. mucosus DFL 24	unknown	Wagner-Döbler 2005												=				=
P. denitrificans	unknown	Schaefer 2002														•		
Oceanibulbus indolifex	unknown	Wagner-Döbler 2005														=		
Staleya guttiformis LM 09	unknown	Wagner-Döbler 2005														• =		
D. shibae DFL 27, 30, 31	unknown	Wagner-Döbler 2005																=
R. mucosus DFL 35	unknown	Wagner-Döbler 2005																=
D. shibae DFL 27, 30, 31, 36	unknown	Wagner-Döbler 2005																==

References:

Ahlgren, N., Harwood, C., Schaefer, A., Giraud, E., and Greenberg, E. (2011). Aryl-homoserine lactone quorum sensing in stem-nodulating photosynthetic bradyrhizobia. Proc. Natl. Acad. Sci. 108, 7183–8. doi:10.1073/pnas.1103821108.

Case, R. J., Labbate, M., and Kjelleberg, S. (2008). AHL-driven quorum-sensing circuits: their frequency and function among the Proteobacteria. ISME J. 2, 345–9. doi:10.1038/ismej.2008.13.

Chandler, J., Duerkop, B., Hinz, A., West, T., Herman, J., Churchill, M., Skerrett, S., and Greenberg, E. (2009). Mutational Analysis of Burkholderia thailandensis Quorum Sensing and Self-Aggregation. J. Bacteriol. 191, 5901–9. doi:10.1128/JB.00591-09.

Conway, B., and Greenberg, E. (2002). Quorum-Sensing Signals and Quorum-Sensing Genes in Burkholderia vietnamiensis. J. Bacteriol. 184, 1187–91. doi:10.1128/JB.184.4.1187–1191.2002.

Dulla, G. F. J., and Lindow, S. E. (2009). Acyl-homoserine lactone-mediated cross talk among epiphytic bacteria modulates behavior of Pseudomonas syringae on leaves. ISME J. 3, 825–34. doi:10.1038/ismej.2009.30.

Eberhard, A., Burlingame, A. L., Eberhard, C., Kenyon, G. L., Nealson, K. H., and Oppenheimer, N. J. (1981). Structural Identification of Autoinducer of Photobacterium fischeri. Biochemistry 20, 2444–2449.

Fuqua, C., and Greenberg, E. P. (2002). Listening in on bacteria: acyl-homoserine lactone signalling. Nat. Rev. Mol. Cell Biol. 3, 685–95. doi:10.1038/nrm907.

Galloway, W. R. J. D., Hodgkinson, J. T., Bowden, S. D., Welch, M., and Spring, D. R. (2011). Quorum Sensing in Gram-Negative Bacteria: Small-Molecule Modulation of AHL and AI-2 Quorum Sensing Pathways. Chem. Rev. 111, 28–67. doi:10.1021/cr100109t.

Iida, A., Ohnishi, Y., and Horinouchi, S. (2008). Control of acetic acid fermentation by quorum sensing via N-acylhomoserine lactones in Gluconacetobacter intermedius. J. Bacteriol. 190, 2546–55. doi:10.1128/JB.01698-07.

Khajanchi, B. K., Kirtley, M. L., Brackman, S. M., and Chopra, A. K. (2011). Immunomodulatory and protective roles of quorum-sensing signaling molecules N-acyl homoserine lactones during infection of mice with Aeromonas hydrophila. Infect. Immun. 79, 2646–57. doi:10.1128/IAI.00096-11.

Lewenza, S., Conway, B., EP, G., and PA, S. (1999). Quorum Sensing in Burkholderia cepacia-Identification of the LuxRI Homologs CepRI. J. Bacteriol. 181, 748–56. Available at: http://jb.asm.org/content/181/3/748.short [Accessed December 9, 2014].

Licciardello, G., Bertani, I., Steindler, L., Bella, P., Venturi, V., and Catara, V. (2007). Pseudomonas corrugata contains a conserved N-acyl homoserine lactone quorum sensing system; its role in tomato pathogenicity and tobacco hypersensitivity response. FEMS Microbiol. Ecol. 61, 222–34. doi:10.1111/j.1574-6941.2007.00338.x.

Llamas, I., Quesada, E., Martínez-Cánovas, M. J., Gronquist, M., Eberhard, A., and González, J. E. (2005). Quorum sensing in halophilic bacteria: detection of N-acyl-homoserine lactones in the

exopolysaccharide-producing species of Halomonas. Extremophiles 9, 333–41. doi:10.1007/s00792-005-0448-1.

Malott, R. J., Baldwin, A., Mahenthiralingam, E., and Sokol, P. A. (2005). Characterization of the cciIR Quorum-Sensing System in Burkholderia cenocepacia Characterization of the cciIR Quorum-Sensing System in Burkholderia cenocepacia. Infect. Immun. 73, 4982–92. doi:10.1128/IAI.73.8.4982.

Marketon, M. M., Gronquist, M. R., Eberhard, A., and González, J. E. (2002). Characterization of the Sinorhizobium meliloti sinR / sinI Locus and the Production of Novel N-Acyl Homoserine Lactones Characterization of the Sinorhizobium meliloti sinR / sinI Locus and the Production of Novel N -Acyl Homoserine Lactones. J. Bacteriol. 184, 5686–95. doi:10.1128/JB.184.20.5686.

Mastroleo, F., Van Houdt, R., Atkinson, S., Mergeay, M., Hendrickx, L., Wattiez, R., and Leys, N. (2013). Modelled microgravity cultivation modulates N-acylhomoserine lactone production in Rhodospirillum rubrum S1H independently of cell density. Microbiology 159, 2456–66. doi:10.1099/mic.0.066415-0.

Mcclean, K. H., Winson, M. K., Fish, L., Taylor, A., Chhabra, S. R., Camara, M., Daykin, M., Lamb, J. H., Swift, S., Bycroft, B. W., et al. (1997). Quorum sensing and Chromobacterium violaceum: exploitation of violacein production and inhibition for the detection of N-acyl homoserine lactones. Microbiology 143, 3703–3711. doi:10.1099/00221287-143-12-3703.

Moré, M. I., Finger, L. D., Stryker, J. L., Fuqua, C., Eberhard, A., and Winans, S. C. (1996). Enzymatic Synthesis of a Quorum-Sensing Autoinducer Through Use of Defined Substrates. Science (80-.). 272, 1655–1658. doi:10.1126/science.272.5268.1655.

Morohoshi, T., Nakamura, Y., Yamazaki, G., Ishida, A., N, K., and Ikeda, T. (2007). The Plant Pathogen Pantoea ananatis Produces N-Acylhomoserine Lactone and Causes Center Rot Disease of Onion by Quorum Sensing. J. Bacteriol. 189, 8333–8. doi:10.1128/JB.01054-07.

Nasuno, E., Kimura, N., Fujita, M. J., Nakatsu, C. H., Kamagata, Y., and Hanada, S. (2012). Phylogenetically novel LuxI/LuxR-type quorum sensing systems isolated using a metagenomic approach. Appl. Environ. Microbiol. 78, 8067–74. doi:10.1128/AEM.01442-12.

Niu, C., Clemmer, K., Bonomo, R., and Rather, P. (2008). Isolation and Characterization of an Autoinducer Synthase from Acinetobacter baumannii. J. Bacteriol. 190, 3386–92. doi:10.1128/JB.01929-07.

Pontes, M., Babst, M., Lochhead, R., Oakeson, K., Smith, K., and Dale, C. (2008). Quorum Sensing Primes the Oxidative Stress Response in the Insect Endosymbiont, Sodalis glossinidius. PLoS One 3, e3541. doi:10.1371/journal.pone.0003541.

Rodelas, B., Lithgow, J., Wisniewski-Dye, F., Hardman, A., Wilkinson, A., Economou, A., Williams, P., and JA, D. (1999). Analysis of quorum-sensing-dependent control of rhizosphere-expressed (rhi) genes in Rhizobium leguminosarum bv. viciae. J. Bacteriol. 181, 3816–23. Available at: http://jb.asm.org/content/181/12/3816.short [Accessed December 9, 2014].

Schaefer, A. L., Greenberg, E. P., Oliver, C. M., Oda, Y., Huang, J. J., Bittan-Banin, G., Peres, C. M., Schmidt, S., Juhaszova, K., Sufrin, J. R., et al. (2008). A new class of homoserine lactone quorum-sensing signals. Nature 454, 595–9. doi:10.1038/nature07088.

Schaefer, A., Taylor, T., Beatty, J., and Greenberg, E. (2002). Long-Chain Acyl-Homoserine Lactone Quorum-Sensing Regulation of Rhodobacter capsulatus Gene Transfer Agent Production. J. Bacteriol. 184, 6515–6521. doi:10.1128/JB.184.23.6515-6521.2002.

Schripsema, J. A. N., de Rudder, K. E., van Vliet, T. B., Lankhorst, P. P., de Vroom, E., Kijne, J. W., and van Brussel, A. A. (1996). Bacteriocin small of Rhizobium leguminosarum belongs to the class of N-acyl-L-homoserine lactone molecules, known as autoinducers and as quorum sensing co-transcription factors. J. Bacteriol. 178, 366–71.

Tahrioui, A., Quesada, E., and Llamas, I. (2011). The hanR/hanI quorum-sensing system of Halomonas anticariensis, a moderately halophilic bacterium. Microbiology 157, 3378–87. doi:10.1099/mic.0.052167-0.

Ulrich, R. L., Deshazer, D., Brueggemann, E., Hines, H., Oyston, P., and Jeddeloh, J. (2004). Role of quorum sensing in the pathogenicity of Burkholderia pseudomallei. J. Med. Microbiol. 53, 1053–1064. doi:10.1099/jmm.0.45661-0.

Vial, L., Cuny, C., Gluchoff-Fiasson, K., Comte, G., Oger, P. M., Faure, D., Dessaux, Y., Bally, R., and Wisniewski-Dyé, F. (2006). N-acyl-homoserine lactone-mediated quorum-sensing in Azospirillum: an exception rather than a rule. FEMS Microbiol. Ecol. 58, 155–68. doi:10.1111/j.1574-6941.2006.00153.x.

Wagner-Döbler, I., Thiel, V., Eberl, L., Allgaier, M., Bodor, A., Meyer, S., Ebner, S., Hennig, A., Pukall, R., and Schulz, S. (2005). Discovery of complex mixtures of novel long-chain quorum sensing signals in free-living and host-associated marine alphaproteobacteria. Chembiochem 6, 2195–206. doi:10.1002/cbic.200500189.
